# A Novel Role of IGF1 in Apo2L/TRAIL-Mediated Apoptosis of Ewing Tumor Cells

**DOI:** 10.1155/2012/782970

**Published:** 2012-10-03

**Authors:** Frans van Valen, Henning Harrer, Marc Hotfilder, Uta Dirksen, Thomas Pap, George Gosheger, Hans-Ulrich Humpf, Heribert Jürgens

**Affiliations:** ^1^Institute of Experimental Musculoskeletal Medicine, University Hospital Münster, Albert-Schweitzer-Campus 1, 48149 Münster, Germany; ^2^Institute of Food Chemistry, The University of Münster, Corrensstrasse 45, 48149 Münster, Germany; ^3^Department of Pediatric Hematology and Oncology, University Children's Hospital Münster, Albert-Schweitzer-Campus 1, 48149 Münster, Germany; ^4^Department of Orthopedic Surgery, University Hospital Münster, Albert-Schweitzer-Campus 1, 48149 Münster, Germany

## Abstract

Insulin-like growth factor 1 (IGF1) reputedly opposes chemotoxicity in Ewing sarcoma family of tumor (ESFT) cells. However, the effect of IGF1 on apoptosis induced by apoptosis ligand 2 (Apo2L)/tumor necrosis factor (TNF-) related apoptosis-inducing ligand (TRAIL) remains to be established. We find that opposite to the partial survival effect of short-term IGF1 treatment, long-term IGF1 treatment amplified Apo2L/TRAIL-induced apoptosis in Apo2L/TRAIL-sensitive but not resistant ESFT cell lines. Remarkably, the specific IGF1 receptor (IGF1R) antibody *α*-IR3 was functionally equivalent to IGF1. Short-term IGF1 incubation of cells stimulated survival kinase AKT and increased X-linked inhibitor of apoptosis (XIAP) protein which was associated with Apo2L/TRAIL resistance. In contrast, long-term IGF1 incubation resulted in repression of XIAP protein through ceramide (Cer) formation derived from de novo synthesis which was associated with Apo2L/TRAIL sensitization. Addition of ceramide synthase (CerS) inhibitor fumonisin B1 during long-term IGF1 treatment reduced XIAP repression and Apo2L/TRAIL-induced apoptosis. Noteworthy, the resistance to conventional chemotherapeutic agents was maintained in cells following chronic IGF1 treatment. Overall, the results suggest that chronic IGF1 treatment renders ESFT cells susceptible to Apo2L/TRAIL-induced apoptosis and may have important implications for the biology as well as the clinical management of refractory ESFT.

## 1. Introduction

The Ewing sarcoma family of tumor (ESFT) of bone and soft tissue includes Ewing sarcoma, peripheral primitive neuroectodermal tumor, and Askin tumor which are among the most aggressive of pediatric malignancies. Despite multidrug chemotherapy in combination with surgery and/or radiation, the long-term survival rate of patients with metastatic and recurrent disease is still dismal [1]. Improved insight into mechanisms of malignancy remains a high priority in ESFT research and is a prerequisite for the identification of new targeted therapies.

Several lines of evidence suggest that IGF1 plays critical roles in the proliferation, metastasis, and survival of ESFT [2]. Specific high-affinity type 1 and type 2 IGF receptors (IGF1R and IGF2R) are expressed in ESFT cells, and metabolic actions such as stimulation of glucose transport, glycogen synthesis, and thymidine incorporation into DNA by both IGF1 and IGF2 are transmitted through IGF1R [3]. An IGF1-IGF1R autocrine circuit was suggested in some ESFT cells and addition of IGF1R antibody caused inhibition of cell proliferation [4]. IGF-binding protein 3 (IGFBP3) production is repressed in ESFT cells by oncogenic fusion proteins, which may allow for IGF1R activation through increased ligand binding [5]. Importantly, IGF1 renders ESFT cells resistant to chemotherapeutic agents [6, 7]. Hofbauer et al. [6] noticed that serum IGF1 is responsible for the induction of chemoresistance in ESFT cells in vitro. Toretsky et al. [7] reported that human recombinant IGF1 can protect ESFT cells from chemotherapy-induced cell death through activation of AKT, a phosphatidylinositol 3-kinase (PI3K-) regulated serine/threonine survival kinase. However, there have been no studies examining the activity of IGF1 in ESFT cells in response to treatment with Apo2L/TRAIL.

Apo2L/TRAIL is a member of the TNF superfamily of cytokines and represents a most valuable candidate for cancer therapy because it initiates apoptosis preferably in tumor cells over normal cells, both in vitro and in vivo in nonhuman primates and mice [8–10]. Moreover, Apo2L/TRAIL is produced by different immune cells and considered as the prime host-defence mechanism against incipient cancers [11]. Clinical studies are ongoing to evaluate the safety and antitumor activity of Apo2L/TRAIL [12]. To date, the cells most susceptible to Apo2L/TRAIL originate from ESFT with apoptotic responsiveness demonstrated in 32 of 40 (80%) established cell lines in vitro and 3 of 3 (100%) patient-derived cells ex vivo thus indicating the eminence of this therapeutic concept [13]. Apo2L/TRAIL triggers apoptosis by interacting with specific death receptors (TRAILR1/DR4 and/or TRAILR2/DR5) followed by activation of initiator caspase-8 and subsequent activation of effector caspases 3 and 7 (extrinsic or death receptor pathway of apoptosis) [14]. Apo2L/TRAIL can also initiate caspase-8-dependent mitochondrial dysfunction leading to activation of caspase-9 (intrinsic or mitochondrial pathway of apoptosis). Intrinsic apoptosis can be regulated negatively by mitochondria-associated antiapoptotic members of the Bcl-2 family, whereas extrinsic apoptosis is primarily blocked by inhibitor of apoptosis protein (IAP) family members including XIAP which binds directly to the catalytically active sites of effector caspases [15, 16]. XIAP is a direct downstream target of AKT and an important mediator of IGF1 on cell survival [17, 18]. Increased levels of XIAP protein and/or AKT activity have been correlated with resistance to Apo2L/TRAIL in various cancer cell types [19–22].

In the present study, we investigated the role of IGF1 in regulating apoptosis induction by Apo2L/TRAIL in ESFT cells. We show that depending on treatment duration IGF1 initially acts as antiapoptotic factor and subsequently as proapoptotic factor in the context of Apo2L/TRAIL lethality. Remarkably, IGF1R antibody *α*-IR3 showed IGF1 mimicry. Moreover, we present the novel finding that IGF1 can amplify Apo2L/TRAIL-induced apoptosis by XIAP repression through de novo ceramide (Cer) formation. This new insight into the action of IGF1 may provide intriguing leads for developing innovative combinatorial approaches for the treatment of refractory ESFT.

## 2. Materials and Methods

### 2.1. Reagents

IGF1 (PeproTech, Rocky Hill, NJ), *α*-IR3, doxorubicin, z-VAD-fmk, z-DEVD-fmk, wortmannin, AKTi-1/2 (AKT inhibitor VIII; Merck Chemicals, Darmstadt, Germany), fumonisin B1 (Biomol Hamburg, Germany), and fatty acid-free bovine serum albumin (Sigma-Aldrich, Taufkirchen, Germany) were purchased from the indicated suppliers. The sphingolipids d17:0-sphinganine, d17:1-sphingosine, d18:1/C17:0-Cer, and d18:1/C17:0-sphingomyelin were obtained from Avanti Polar Lipids (Alabaster, AL, USA). Etoposide was generously supplied by Cipla (Mumbai, India) through Medac (Wedel, Germany). Apo2L/TRAIL was generously provided by Genentech (San Francisco, CA, USA).

### 2.2. Cell Culture and Incubation Conditions

The following human ESFT cell lines were studied: A9423 (provided by Timothy Triche, Los Angeles, CA, USA), ES-2 (provided by Thomas Look, Memphis, TN, USA), LAP-35 (provided by Katia Scotlandi, Bologna, Italy), and A17/95 (provided by Ursula Anderer, Berlin, Germany). ESFT cell lines RD-ES and SK-ES-1 were obtained from the ATCC (Rockville, MD, USA), and CADO-ES-1 and MHH-ES-1 were from the German Collection of Microorganisms and Cell Cultures (Braunschweig, Germany). ESFT cell lines VH-64 and WE-68 were previously established by one of us (F.v.V.) [3]. The clinical data and the cytogenetic features of all cell lines have been reviewed [23]. Cells were maintained in RPMI-1640 medium supplemented with 10% foetal calf serum (FCS), 1% antibiotic antimycotic mixture (10 mg/mL streptomycin, 10,000 U/mL penicillin and 25 *μ*g/mL amphotericin B), and 2 mM L-glutamine (Gibco, Grand Island, NY, USA) (i.e., serum-containing medium) at 37°C in a humidified atmosphere with 5% CO_2_. Cells were subcultured in 25 cm^2^ tissue culture flasks using 0.05% trypsin/0.02% EDTA in Puck's saline (Gibco). Cells (8 × 10^4^/cm^2^) were seeded in 96-well plates for cell viability, in white-walled 96-well luminometer plates for caspase assays, in 12-well plates for apoptosis analysis, in 6-well plates for protein extraction, and in 75-cm^2^ tissue culture flasks for sphingolipid extraction. All synthetic culture ware (Falcon Plastics, Oxnard, CA, USA) was collagen-coated (5 *μ*g/cm^2^; BioConcept, Salem, NH, USA) as described [13]. For analysis of the effects of IGF1, cultures were washed and maintained in serum-free medium substituting serum by 0.2% fatty acid-free bovine serum albumin (BSA; Sigma-Aldrich, Taufkirchen, Germany) for 24 hours until experimentation. Cells were pretreated with IGF1 followed by treatment with Apo2L/TRAIL for the indicated times. To assess the effects of pharmacological inhibitors, these agents were added to the cultures at the indicated concentrations 30 min prior to treatment with IGF1 and/or Apo2L/TRAIL.

### 2.3. Apoptosis and Cell Viability Assays

For fluorescence-activated cell sorting (FACS) analysis of apoptosis, cells were detached with 5 mM EDTA in PBS and washed in PBS containing 0.2% BSA. Cells were incubated with R-phycoerythrin-conjugated annexin-V (1 : 100; Invitrogen, Darmstadt, Germany) and 7-amino-actinomycin D (7-AAD, 1 : 200; Invitrogen) at room temperature in the dark. After 20 min cells were washed in ice-cold PBS containing 0.5% sodium azide and analyzed by FACS-Calibur (BD Biosciences, Heidelberg, Germany) using CellQuest Pro software (BD Biosciences). Specific apoptosis was calculated as follows: 100 × [experimental apoptosis (%) − spontaneous apoptosis (%)/100%  − spontaneous apoptosis (%)]. For assessment of cell viability, the spectrophotometric conversion of 3-(4,5-dimethylthiazol-2-yl)-2,5-diphenyltetrazolium bromide (MTT; Sigma-Aldrich) was measured using a Victor^3^ 1420 Multilabel Counter (PerkinElmer, Rodgau, Germany) at 550 nm. Percentage of cell viability was calculated by the formula 100 × (absorbance of experimental wells/absorbance of control wells).

### 2.4. Caspase Assays

Casapase-8, caspase-9, and capase-3/7 activities were analyzed by the Caspase-Glo-8, Caspase-Glo-9, respectively, Caspase-Glo-3/7 assays (Promega, Madison, WI, USA). Cells were exposed to IGF1 for the times indicated and subsequently incubated for 4 hours with Apo2L/TRAIL. Thereafter, an equal volume of Caspase-Glo-8, Caspase-Glo-9, or Caspase-Glo-3/7 reagent was added to the cells and cultures were incubated for an additional 45 min. The aminoluciferin released by caspase-mediated cleavage of proluminogenic substrate was determined using Victor^3^ plate reader (PerkinElmer). Background luminescence was determined using reactions-lacking cells. Caspase activity was expressed as fold increase in relative light unit ratio between the caspase activities of treated cells versus untreated cells (normalized to relative unit of 1.0).

### 2.5. Sphingolipid Identification and Quantitation

Cells were harvested by trypsinization, washed in ice-cold PBS and pelleted, and stored under liquid nitrogen. For lipid extraction, cellular pellets were lyophilized and extracted using a mixture of ethyl acetate : isopropanol : water (60 : 30 : 10, v/v/v) [24]. The samples were spiked with a sphingolipid internal standard mixture consisting of d17:0-sphinganine (50 pmol), d17:1-sphingosine (150 pmol), d18:1/C17:0-Cer (250 pmol), and d18:1/C17:0-sphingomyelin (500 pmol). Sphingolipids were quantitated by high-performance liquid chromatography coupled to electrospray ionization tandem mass spectrometry (HPLC-ESI-MS/MS) on an API 4000 triple quadrupole mass spectrometer (Applied Biosystems, Darmstadt, Germany). Cellular sphingolipid levels were normalized to viable cell number.

### 2.6. Western Blot Analysis

Harvested cells were rinsed in PBS containing 100 *μ*M Na_3_VO_4_ and lyzed for 30 min at 4°C in Triton X-100 buffer (20 mM Tris, pH 7.5, 150 mM NaCl, 1 mM EDTA, 1 mM EGTA, 2.5 mM sodium pyrophosphate, 1 mM *β*-glycerophosphate, 1 mM Na_3_VO_4_, 1 *μ*g/mL leupeptin, 1 mM PMS, 1% Triton X-100). Protein expression was determined in whole-cell lysates (50–100 *μ*g) using detergent compatible Bio-Rad protein assay. Proteins were dissolved in Laemmli buffer, resolved on 12% SDS-PAGE gels, and transferred to PVDF. Membranes were blocked with 5% Western blot blocking buffer and incubated with the appropriate antibodies. Primary antibodies used were rabbit anti-AKT polyclonal antibody (1 : 500), rabbit anti-pAKT(Ser473) polyclonal antibody (1 : 100), rabbit anti-Mcl-1 polyclonal antibody (1 : 500; Santa Cruz Biotechnology, Santa Cruz, CA, USA), mouse anti-XIAP mAb (1 : 250), and rabbit anti-Bax polyclonal antibody (1 : 1,000; BD Biosciences Pharmingen, Heidelberg, Germany). The secondary antibodies applied were goat anti-mouse IgG (1 : 5,000; DakoCytomation, Hamburg, Germany) and goat anti-rabbit IgG (1 : 5,000; DakoCytomation). Enhanced chemiluminescence (GE Healthcare, Freiburg, Germany) was employed for signal detection. Unless otherwise indicated, equal protein loading was monitored by immunodetection of *β*-actin using mouse anti-*β*-actin mAb (1 : 25,000; Sigma-Aldrich).

### 2.7. Immunoassay Evaluation of Proteins

Expression of pAKT(Ser473) and total AKT in cell lysates was validated by PathScan Sandwich enzyme-linked immunosorbent assay (ELISA; Cell Signaling Technology, Beverly, MA, USA). Quantitative determination of XIAP was conducted by TiterZyme enzyme immunometric assay (EIA; Assay Designs, Ann Arbor, MI, USA). Cell lysis buffer was supplemented with protease inhibitor cocktail (P-8340; Sigma-Aldrich). Optical density was determined at 450 and 570 nm using Victor^3^ plate reader (PerkinElmer). Data were expressed as pAKT/total AKT ratio. XIAP protein concentration was expressed as nanogram per 10^6^ viable cells. Number of viable cells was determined using CASY Counter (Roche Applied Science, Penzberg, Germany) per the manufacturer's protocol.

### 2.8. Statistical Analysis

Differences between experimental groups were evaluated by ANOVA and Tukey's *post-hoc* test using SigmaStat 3.5 software (Systat Software Inc., San Jose, CA, USA). A *P* value < 0.05 was regarded as statistically significant.

## 3. Results

**Figure 1:**
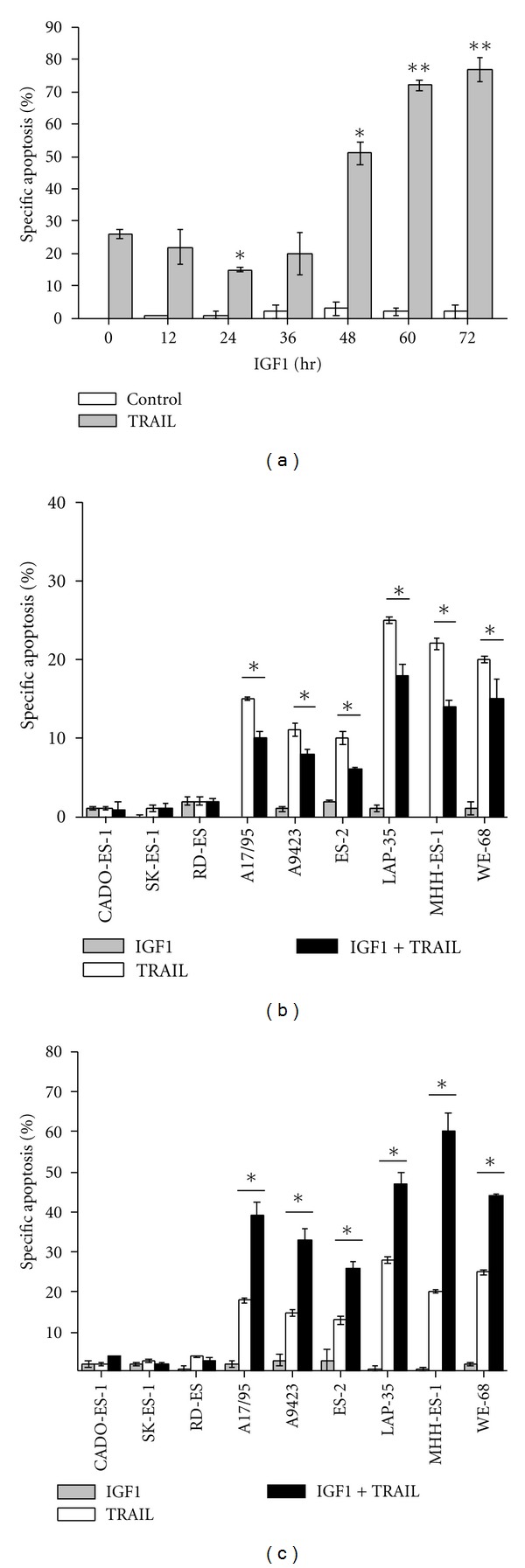
Time-dependency of IGF1 on Apo2L/TRAIL-induced apoptosis. (a) Percentage-specific apoptosis in VH-64 cells treated for the times indicated with IGF1 (50 ng/mL), followed by incubation with Apo2L/TRAIL (TRAIL; 50 ng/mL; 8 hours) or serum-free medium alone (Control). Percentage-specific apoptosis in cells from different ESFT lines treated for (b) 24 hours and (c) 72 hours in the absence and presence of IGF1 (50 ng/mL), followed by incubation with Apo2L/TRAIL (TRAIL; 50 ng/mL; 8 hours) or serum-free medium alone. Asterisks: *P < 0.05; **P < 0.02; error bars: mean ± SD of triplicate determinations from 3 independent experiments.

We first examined the temporal effect of IGF1 on Apo2L/TRAIL lethality in ESFT cells. As revealed in Figure 1(a), incubation of VH-64 cells with 50 ng/mL IGF1 resulted in gradual protection from apoptosis induced by 50 ng/mL Apo2L/TRAIL, with significant resistance observed 24 hours after IGF1 addition. However, as time of incubation continued, IGF1 caused a marked amplification of apoptosis induced by Apo2L/TRAIL, beginning 48 hours after IGF1 addition. After 72 hours, IGF1 elicited a 3-fold increase in the percentage of apoptotic cells compared with untreated control. IGF1 as single agent did not induce apoptosis in VH-64 cells, not even when cells were exposed for up to 72 hours with IGF1 (Figure 1(a)).

Besides cell line VH-64, additional ESFT cell lines also displayed time-dependent dual regulation of Apo2L/TRAIL sensitivity by IGF1. The results are summarized in Figures 1(b) and 1(c). While 24 hours of IGF1 treatment caused suppression of Apo2L/TRAIL lethality (Figure 1(b)), 72 hours of IGF1 treatment caused amplification of Apo2L/TRAIL lethality (Figure 1(c)) in ESFT cell lines A17/95, A9423, ES-2, LAP-35, MHH-ES-1, and WE-68, which like VH-64 are all sensitive to Apo2L/TRAIL [13]. In contrast, IGF1 treatment for 24 hours and 72 hours did not affect the Apo2L/TRAIL response in ESFT cell lines CADO-ES-1, SK-ES-l, and RD-ES (Figures 1(b) and 1(c)), which are all resistant to Apo2L/TRAIL [13]. IGF1 (100 ng/mL) alone provoked minimal, if at all, apoptosis in the ESFT cell lines examined (Figures 1(b) and 1(c)). These results suggest that depending on treatment duration IGF1 initially inhibits apoptosis and subsequently promotes apoptosis induced by Apo2L/TRAIL.

**Figure 2:**
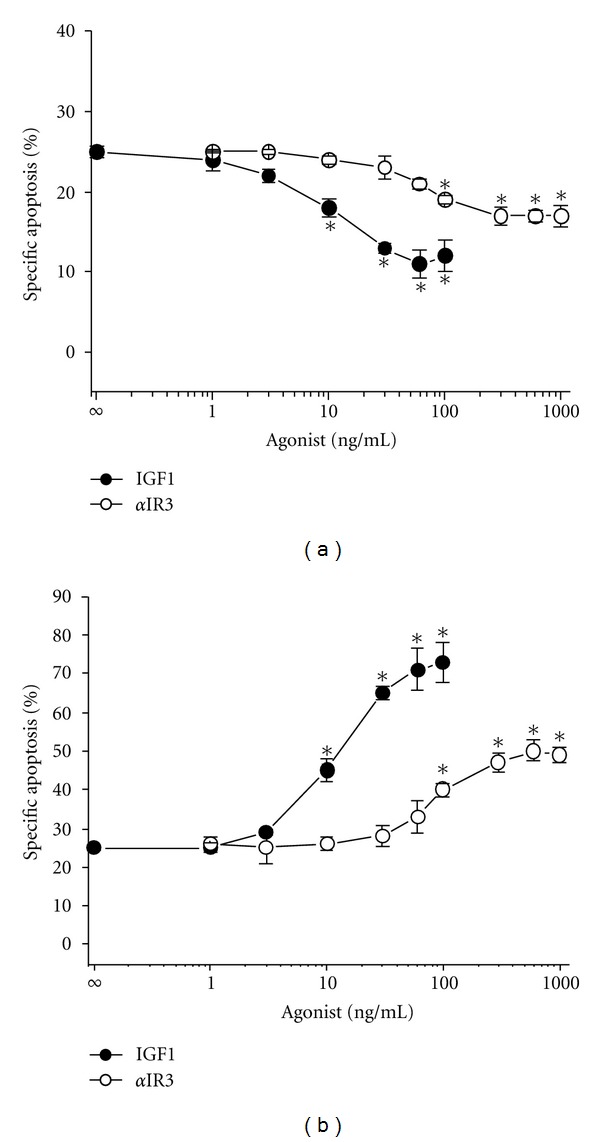
Concentration dependency of IGF1 and *α*-IR3 on Apo2L/TRAIL-induced apoptosis. Percentage-specific apoptosis in VH-64 cells treated for (a) 24 hours and (b) 72 hours with the indicated concentrations of IGF1 and *α*-IR3, followed by incubation with Apo2L/TRAIL (50 ng/mL; 8 hours). All incubations contained nonspecific isotype IgG at a final concentration of 3 *μ*g/mL. Asterisks: *P < 0.05; error bars: mean ± SD of triplicate determinations from 3 independent experiments.

Next, IGF1 concentration response experiments were performed. As shown in Figure 2(a), IGF1 in the range 3–100 ng/mL induced apoptosis resistance to Apo2L/TRAIL in VH-64 cells with IC_50_ ~ 10 ng/mL of IGF1 during 24-hour incubation periods. Equivalent concentrations of IGF1 (3–100 ng/mL) and IC_50_ (~10 ng/mL IGF1) amplified Apo2L/TRAIL lethality during 72-hour incubation periods (Figure 2(b)). The IGF1R monoclonal antibody *α*-IR3 in the range 100–1000 ng/mL also elicited a biphasic effect on Apo2L/TRAIL-induced apoptosis causing suppression during 24-hour incubation periods and amplification during 72-hour incubation periods (Figures 2(a) and 2(b)). Under each incubation condition, the antibody was 10 times less efficient (IC_50_ ~ 100 ng/mL of *α*-IR3) than IGF1. When used at peak concentrations, *α*-IR3 was ~2 times less potent compared with IGF1. This finding suggests that IGF1R-binding sites with similar affinities for agonist are responsible for transduction of the opposite biological responses.

**Figure 3:**
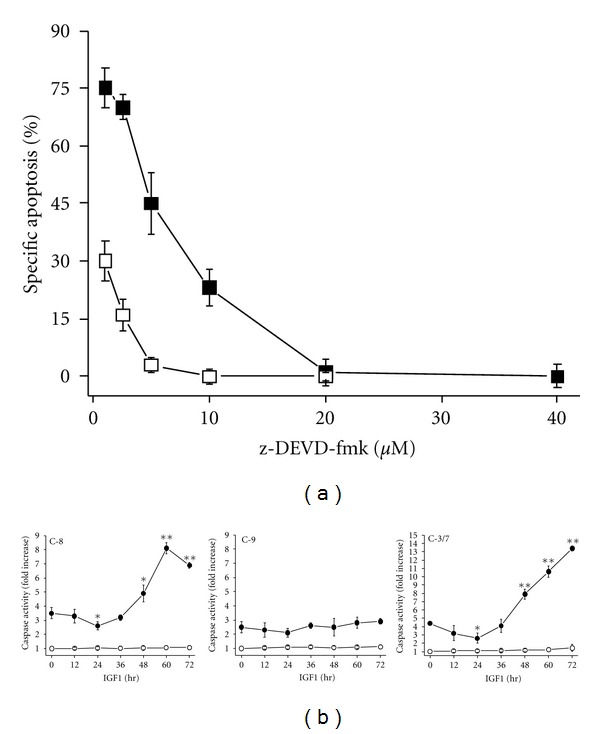
Involvement of caspase-like proteases in Apo2L/TRAIL-induced apoptosis. (a) Percentage-specific apoptosis in VH-64 cells treated for 72 hours in the absence (□) and presence (■) of IGF1 (100 ng/mL) and subsequently for 30 min with varying concentrations of z-DEVD-fmk, followed by incubation with Apo2L/TRAIL (50 ng/ml; 8 hours). Error bars: mean ± SD of triplicate determinations from 2 independent experiments. (b) Fold increase in the activity of caspase-8 (C-8), caspase-9 (C-9), and caspase-3/7 (C-3/7) in cells treated for the indicated times with IGF1 (100 ng/mL), followed by incubation in the absence (°) and presence (●) of Apo2L/TRAIL (50 ng/mL; 4 hours). Values of vehicle-treated controls for caspase-8, caspase-9, and caspase-3/7 activity were set to 1. Asterisks: *P < 0.05, **P < 0.02; error bars: mean ± SD of triplicate determinations from 3 independent experiments.

To get further insight into the mechanism by which IGF1 modulates Apo2L/TRAIL lethality in ESFT cells, we investigated whether caspase-like proteases were involved in Apo2L/TRAIL-induced apoptosis. As shown in Figure 3(a), the caspase-3/7 antagonist z-DEVD-fmk prevented Apo2L/TRAIL lethality in VH-64 cells in a dose-dependent fashion in both IGF1-treated and untreated cells.

We then examined the effect of IGF1 on different components of the caspase cascade. The activation of caspase-8, a key upstream mediator of extrinsic and intrinsic apoptosis, caspase-9, a downstream mediator of intrinsic apoptosis, and caspase-3/7, key effector caspases of extrinsic and intrinsic apoptosis was monitored by luminescence assay. IGF1 incubation of VH-64 cells suppressed the activation of caspase-8 and caspase-3/7 by Apo2L/TRAIL after 24 hours but increased their activities after 48–72 hours (Figure 3(b)). IGF1 failed to modify the activation of caspases-9 by Apo2L/TRAIL. During the entire incubation period, IGF1 itself did not significantly affect caspase activity in VH-64 cells.

**Figure 4:**
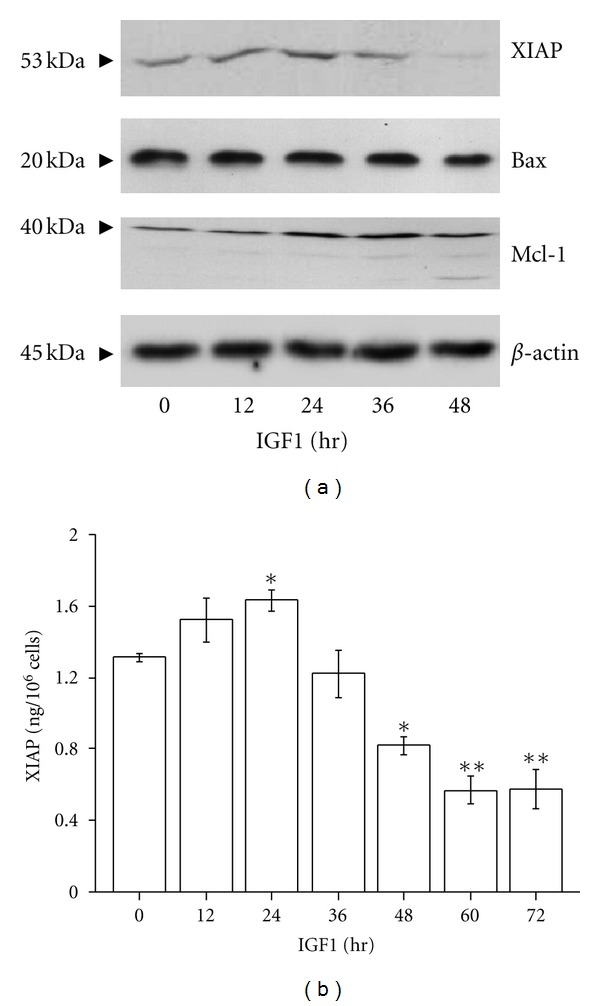
IGF1 has biphasic effects on XIAP protein expression. (a) Western blot assays of VH-64 cell lysates with antibodies to XIAP, Bax, and Mcl-1. Cells were treated for the indicated times with IGF1 (100 ng/mL). Representative nonstripped blots separately probed with the different antibodies are shown. (b) EIA analysis of XIAP in cells incubated for the indicated times with IGF1 (100 ng/mL). Asterisks: *P < 0.05, **P < 0.02; error bars: mean ± SD of triplicate determinations from 4 independent experiments.

We next investigated whether IGF1 could modulate the expression of specific antiapoptotic and proapoptotic proteins that may regulate Apo2L/TRAIL signaling in ESFT cells. Incubation of VH-64 cells with IGF1 stimulated the expression of XIAP protein within 12–24 hours (Figure 4(a)). However, prolonged incubation of cells with IGF1 revealed that XIAP stimulation was only transient and was inhibited 48 hours after IGF1 addition. Mitochondria-related proapoptotic Bax appeared somewhat less at 24–48 hours and antiapoptotic Mcl-1 appeared more at 24–48 hours than at time zero. Immunoassay evaluation of XIAP affirmed that IGF1 exhibited a biphasic effect on XIAP expression: 12–24 hours after IGF1 incubation XIAP protein level was increased, followed by its reduction to below basal level at 48–72 hours (Figure 4(b)).

**Figure 5:**
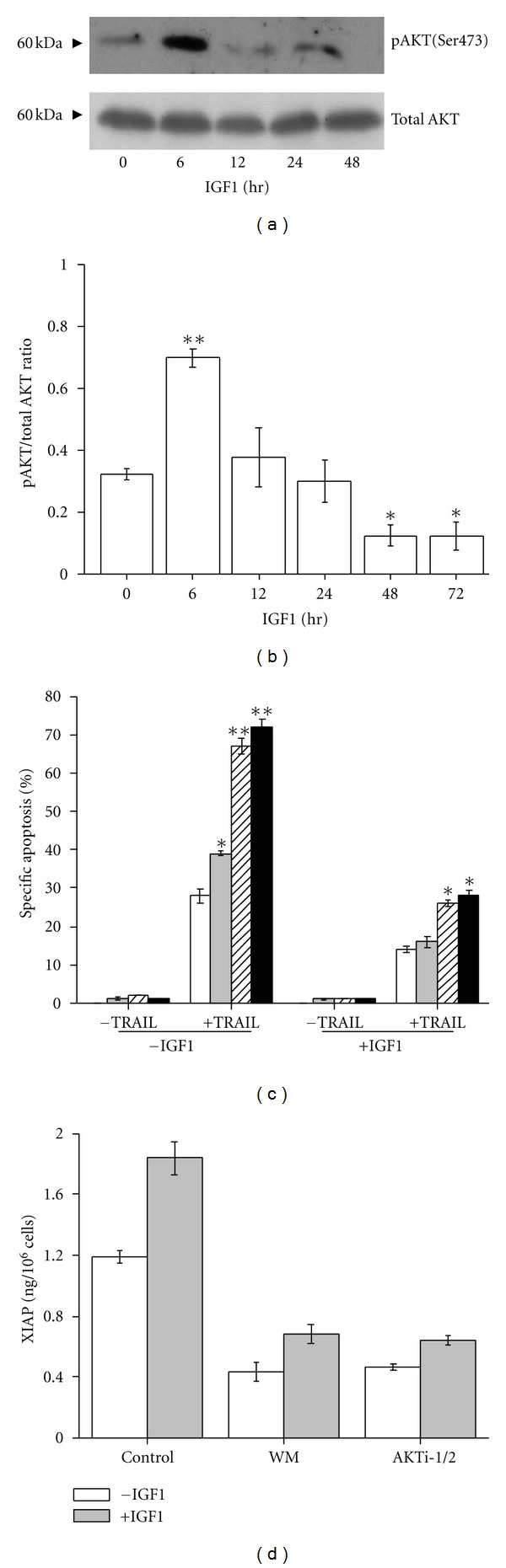
Activation of AKT is associated with increased XIAP protein expression and Apo2L/TRAIL resistance. (a) Western blot assays of VH-64 cell lysates with antibodies to phosphorylated AKT and total AKT. Cells were treated for the indicated times with IGF1 (100 ng/mL). The antitotal AKT blot serves as a loading control. Representative nonstripped blots separately probed with the different antibodies are shown. (b) Ratio of phosphorylated AKT to total AKT determined by ELISA in cells treated for the indicated times with IGF1 (100 ng/mL). (c) Percentage-specific apoptosis in VH-64 cells treated without (white columns) and with 50 nM (grey columns), 200 nM (striped columns), and 500 nM (black columns) wortmannin in the absence and presence of IGF1 (50 ng/mL; 24 hours), followed by incubation without and with Apo2L/TRAIL (TRAIL; 50 ng/mL; 8 hours). (d) EIA analysis of XIAP in cells treated with wortmannin (WM; 500 nM), AKTi-1/2 (2 *μ*M), and serum-free medium alone (Control), followed by incubation in the absence and presence of IGF1 (100 ng/mL; 24 hours). Asterisks: *P < 0.05, **P < 0.02; error bars: mean ± SD of triplicate determinations from 3 independent experiments.

AKT mediates survival of ESFT cells induced by IGF1 [7]. Moreover, Dan et al. [17] reported that XIAP is a direct target of AKT. We first investigated the effect of IGF1 on AKT activity. As shown in Figure 5(a), incubation of VH-64 cells with IGF1 resulted in phosphorylation of pAKT within 6 hours. However, continuous incubation of cells with IGF1 led to gradual inhibition of AKT activity, as demonstrated by the hypophosphorylation of pAKT at 48 hours. pAKT/total AKT ratio analysis also revealed dual regulation of AKT activity by IGF1: 6 hours after IGF1 addition the ratio is increased, followed by its decrease to below basal values at 48–72 hours (Figure 5(b)). Treatment of cells with 500 nM wortmannin, a specific PI3K inhibitor, reduced the basal pAKT/total AKT ratio from 0,321 ± 0,019 to 0,123 ± 0,011, which is comparable to the ratio obtained after incubation of cells for 72 hours with IGF1 (50 ng/mL) alone (i.e., 0,121 ± 0,009). Moreover, wortmannin completely prevented the increase in pAKT/total AKT ratio after treatment of cells with IGF1 for up to 24 hours (data not shown).

We then examined whether inhibition of AKT activity by wortmannin could alter apoptosis induced by Apo2L/TRAIL in ESFT cells. Treatment of VH-64 cells with wortmannin augmented Apo2L/TRAIL-induced apoptosis in a concentration-dependent manner. Sensitization to Apo2L/TRAIL was more efficient in untreated cells, compared to cells that had been treated with IGF1 for 24 hours (Figure 5(c)).

To evaluate the importance of AKT activity in XIAP expression induced by IGF1, we measured XIAP protein levels in VH-64 cells treated with wortmannin and the specific AKT inhibitor AKTi-1/2. As shown in Figure 5(d), wortmannin (500 nM) and AKTi-1/2 (2 *μ*M) reduced constitutive XIAP levels and partially inhibited the stimulation of XIAP induced by IGF1 after 24 hours.

Overall, these results suggest that activation of PI3K-AKT is associated with increased XIAP expression and Apo2L/TRAIL resistance in VH-64 cells in response to acute IGF1 stimulation.

**Figure 6:**
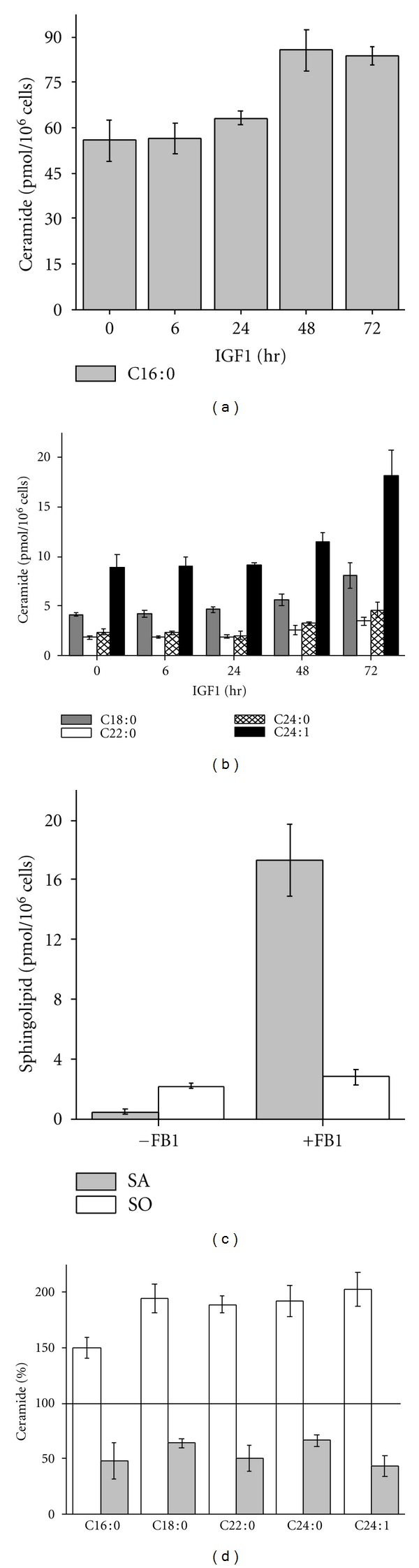
IGF1 increases de novo Cer formation. Levels of (a) C16 : 0-Cer and (b) C18 : 0-, C22 : 0-, C24 : 0-, and C24 : 1-Cer in VH-64 cells incubated for the indicated times with IGF1 (100 ng/mL). (c) Levels of sphinganine (SA) and sphingosine (SO) in VH-64 cells treated for 72 hours with IGF1 (100 ng/mL) in the absence and presence of FB1 (30 *μ*M). (d) Percentage of different Cer species in VH-64 cells treated for 72 hours with IGF1 (100 ng/mL) in the absence (white columns) and presence (grey columns) of FB1 (30 *μ*M). Values of vehicle-treated controls for the different Cer species were set to 100%. Error bars: mean ± SD of triplicate determinations.

The sphingolipid molecule ceramide (Cer) has been implicated in proapoptotic signaling in response to IGF1 [25]. Furthermore, Cer formation has been linked to inhibition of AKT signaling and downregulation of XIAP protein expression [26, 27]. To evaluate the importance of Cer generation in Apo2L/TRAIL sensitization, XIAP suppression and AKT inhibition induced by chronic IGF1 stimulation, we first measured intracellular Cer levels in VH-64 cells treated with IGF1. Several Cer species were detected, particularly C16 : 0-, C18 : 0-, C22 : 0-, C24 : 0-, and C24 : 1-Cer of which C16 : 0-Cer was the most abundant compound determined by HPLC-ESI-MS/MS (Figures 6(a) and 6(b)). Cer levels were not changed by IGF1 within 24 hours. However, treatment of cells with IGF1 for 48–72 hours resulted in 1.5- to 2-fold increase in Cer formation. Intracellular Cer formation is derived from two main pathways: the ceramide synthase (CerS-) dependent Cer de novo pathway and the CerS-dependent Cer salvage pathway [28]. To delineate the pathway that is utilized by IGF1 to increase Cer generation, we measured expression of sphinganine, a precursor in the de novo Cer pathway, and sphingosine, a precursor in the Cer salvage pathway. As revealed in Figure 6(c), addition of the specific CerS inhibitor fumonisin B1 (FB1) during IGF1 treatment resulted in the accumulation of sphinganine but not sphingosine. Moreover, treatment of cells with FB1 completely blocked the stimulation of Cer formation by IGF1 (Figure 6(d)).

**Figure 7:**
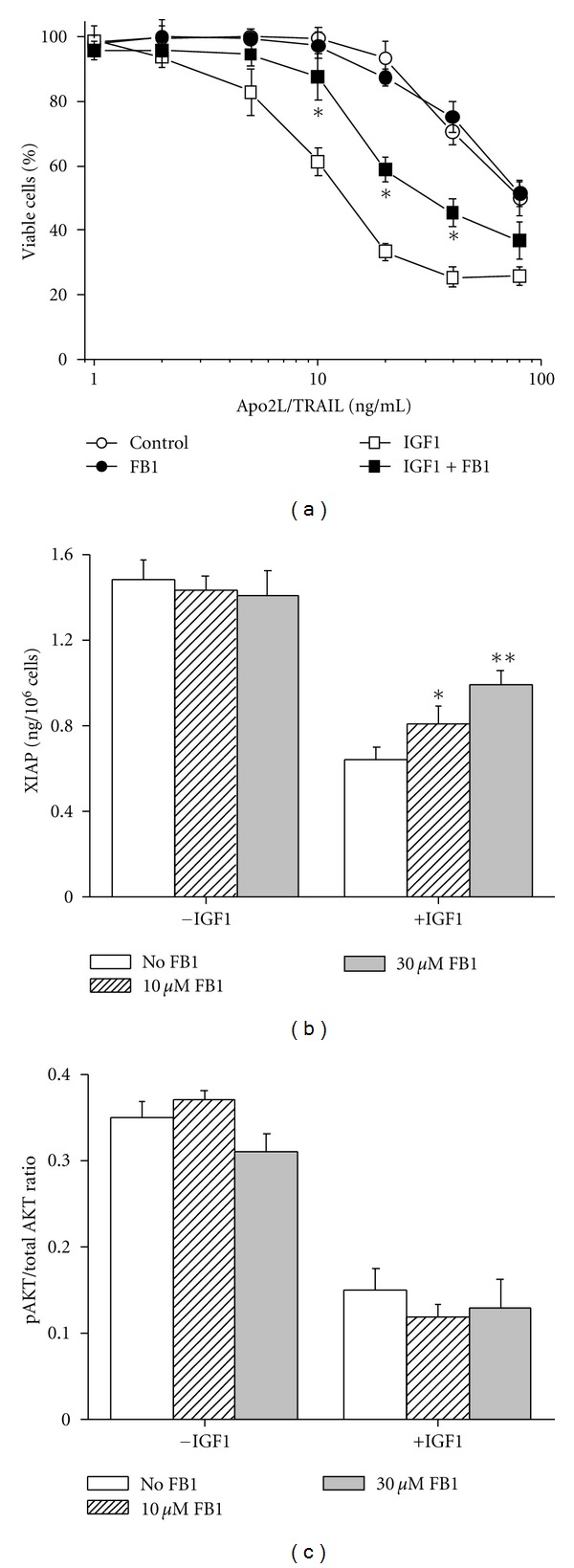
Functional consequences of Cer formation. (a) Percentage of viable VH-64 cells treated for 72 hours without (°, ●) and with (□, ■) IGF1 (100 ng/mL) in the absence (open symbols) and presence (closed symbols) of FB1 (30 *μ*M), followed by incubation for 18 hours with the indicated concentrations of Apo2L/TRAIL. (b) EIA analysis of XIAP in VH-64 cells treated for 72 hours without and with IGF1 (100 ng/mL), in the presence of the indicated concentrations of FB1. (c) Ratio of phosphorylated AKT to total AKT determined by ELISA in VH-64 cells treated for 72 hours without and with IGF1 (100 ng/mL) in presence of the indicated concentrations of FB1. Asterisks: *P < 0.05, **P < 0.02; error bars: mean ± SD of quadruplicate determinations from 3 independent experiments.

We then investigated the functional consequences of Cer generation by IGF1 using FB1. As shown in Figure 7(a), addition of FB1 suppressed Apo2L/TRAIL lethality in IGF1-treated but not untreated cells. To assess whether Cer formation amplifies Apo2L/TRAIL-induced apoptosis through suppression of XIAP levels, we investigated the effect of FB1 on XIAP expression in long-term IGF1-stimulated cells. As demonstrated in Figure 7(b), FB1 reversed the repression of XIAP protein in a concentration-dependent manner in IGF1-treated but not untreated cells. Under these conditions, FB1 did not modulate the inhibition of AKT activity induced by IGF1 after 72 hours (Figure 7(c)).

Overall, these results suggest that Cer formation via de novo synthesis mediates XIAP suppression and Apo2L/TRAIL amplification in VH-64 cells in response to chronic IGF1 stimulation.

**Figure 8:**
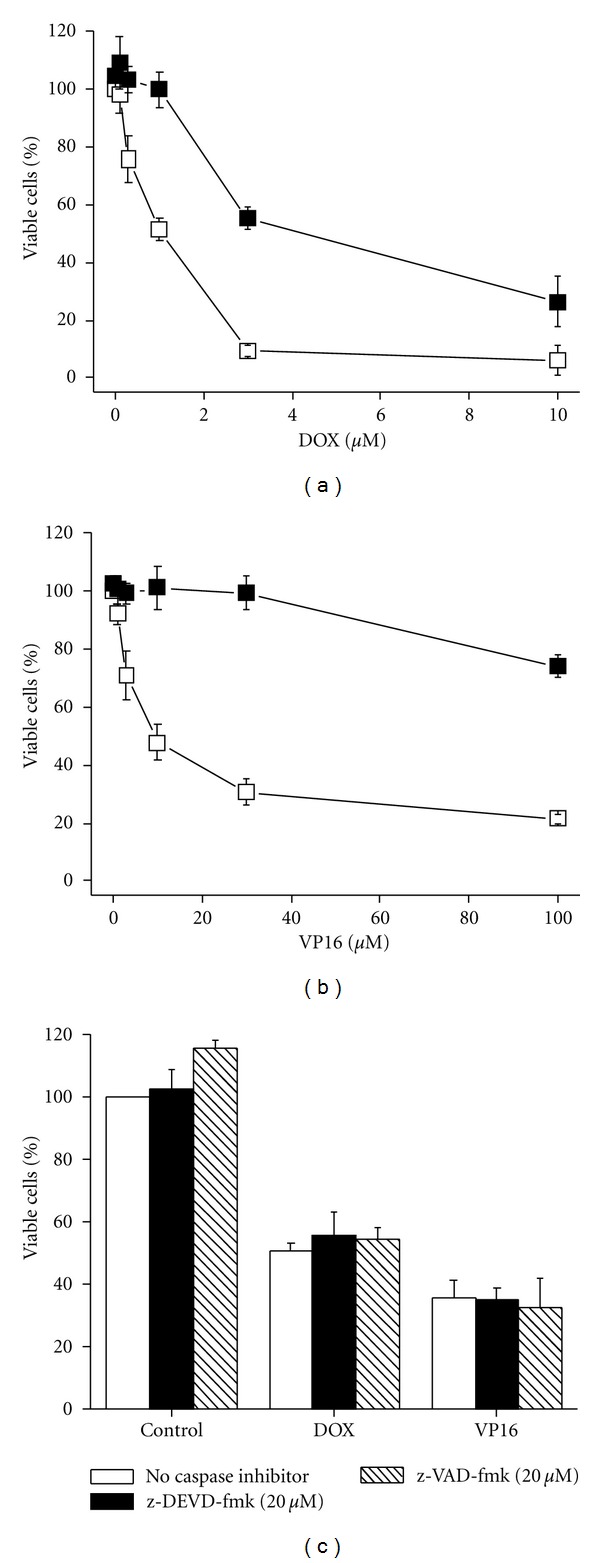
Long-term IGF1 treatment induces resistance to conventional chemotherapeutic agents. Percentage of viable VH-64 cells incubated for 72 hours in the absence (□) and presence (■) of IGF1 (100 ng/mL), followed by incubation for 18 hours with the indicated concentrations of (a) DOX and (b) VP16. (c) Percentage of viable VH-64 cells incubated for 30 min in the absence and presence of 20 *μ*M z-DEVD-fmk and 20 *μ*M z-VAD-fmk, followed by incubation for 18 hours with DOX (1.5 *μ*M), VP16 (20 *μ*M), and serum-free medium alone (Control). Error bars: mean ± SD of triplicate determinations from 4 independent experiments.

Studies in ESFT cells have shown that short-term IGF1 treatment (≤24 hours) provides resistance to doxorubicin (DOX) and etoposide (VP16) [6, 7], and this was confirmed by us (data not shown). We then assessed the effect of chronic IGF1 treatment on chemotoxicity. As shown in Figures 8(a) and 8(b), exposure of VH-64 cells to IGF1 (100 ng/mL) for 72 hours induced resistance to DOX (1–10 *μ*M) and VP16 (10–100 *μ*M). Evaluation of the mode of chemotherapy-induced toxicity revealed that z-DEVD-fmk and the pan-caspase antagonist z-VAD-fmk did not prevent DOX- and VP16-induced cell death (Figure 8(c)).

## 4. Discussion

In this study we investigated the role of IGF1 in regulating the responsiveness of ESFT cells to apoptosis induced by Apo2L/TRAIL. Our investigation revealed that time is an important factor in deciding whether IGF1 may act as survival factor or proapoptotic factor in the context of Apo2L/TRAIL-induced apoptosis. The results show that chronic IGF1 stimulation enhances Apo2L/TRAIL responsiveness of Apo2L/TRAIL-sensitive but not resistant ESFT cell lines, suggesting that IGF1 acts by promoting Apo2L/TRAIL sensitivity rather than by reversing Apo2L/TRAIL resistance *per se*.

Another interesting aspect of the present study relates to *α*-IR3. This IGF1R antibody has been utilized to define the specificity of IGF1 and IGF2 binding to IGF1R in ESFT cells in vitro [3]. *α*-IR3 has also been employed to influence the growth of ESFT cells in vitro and in vivo [4, 29, 30]. The inhibition of cell proliferation by long-term *α*-IR3 treatment has been suggested to result from blockade of IGF1R activation by growth factor produced by the cells in an autocrine fashion. In our experiments, *α*-IR3 behaved as a partial agonist, mimicking the anti- and proapoptotic effects of IGF1 on Apo2L/TRAIL lethality in VH-64 cells. These cells reportedly release minimal amounts of IGF1 (~1–3 ng/10^6^ cells) into serum-depleted culture media [23]. Moreover, under the conditions employed, *α*-IR3 did not affect the growth of VH-64 cells (unpublished data). Concerning *α*-IR3 with agonist characteristic, several studies in normal and malignant cells have demonstrated that *α*-IR3 can stimulate a wide variety of biological events including phosphorylation/activation and downregulation/internalization of IGF1R, release of vascular endothelial growth factor, synthesis of IGFBPs, cell proliferation, apoptosis protection, and apoptosis induction [25, 31–35].

Our results concord with previous accounts of PI3K-AKT-dependent protection against death inducing stimuli in ESFT cells [7, 36]. We have shown that inhibition of PI3K-AKT by wortmannin and AKTi-1/2 results in sensitization to Apo2L/TRAIL, suggesting that PI3 K-AKT signaling prevents efficient induction of apoptosis by Apo2L/TRAIL. We demonstrated that wortmannin and AKTi-1/2 reduce XIAP protein expression. However, wortmannin and AKTi-1/2 did not completely prevent stimulation of XIAP by IGF1, implying that alternative survival signalling pathways are also involved in the stimulation of XIAP protein expression by IGF1. We have shown that incubation with wortmannin for 24 hours does not affect VH-64 cell viability, indicating that AKT inactivation itself does not induce apoptosis. Thus, the findings suggest that increased XIAP protein expression by PI3K-AKT is one mechanism underlying Apo2L/TRAIL resistance in ESFT cells in response to short-term IGF1 stimulation.

We demonstrated that long-term IGF1 treatment induces inactivation of AKT and inhibition of XIAP protein levels in VH-64 cells. Meanwhile, the same treatment results in de novo Cer formation, which correlated well with the progression of Apo2L/TRAIL sensitivity. Although we have shown that AKT activation is associated with XIAP upregulation by early IGF1 stimulation, the inhibition of AKT does not fully account for XIAP downregulation by IGF1 at later stages. We find that de novo-generated Cer mediates repression of XIAP at a level downstream of AKT. The mechanism by which Cer may cause downregulation of XIAP in ESFT cells is presently unknown. Studies in B-cell receptor (BcR-) activated lymphoma cells have shown that de novo Cer formation induces degradation of XIAP in a proteasome-dependent manner [27]. Also, the mechanism for the inactivation of AKT in response to chronic IGF1 stimulation is unclear. Studies have shown that chronic IGF1 stimulation of mammalian target of rapamycin (mTOR) results in feedback inhibition of PI3K-AKT through the phosphorylation and subsequent proteasomal degradation of insulin receptor substrate 2 (IRS2) [37].

In addition to AKT activity and XIAP protein expression, caspase-8 and caspase-3/7 activities are also regulated in a dual manner by IGF1. In contrast, the activation of caspase-9 by Apo2L/TRAIL was not affected even though chronic IGF1 stimulation decreased Bax and increased Mcl-1 expression. Together, these results indicate that the biphasic effect of IGF1 on Apo2L/TRAIL sensitivity relies to a large extent on modulation of the extrinsic rather than the intrinsic pathway of apoptosis.

 Evidence indicates that de novo-generated Cer itself can trigger cell death, for example, by stimulation of caspases [38]. In this study, de novo Cer formation upon treatment of cells for 72 hours with IGF1 did not result in apoptosis. This observation contrasts with Cer-species accumulated de novo in other systems in response to various extracellular factors. For example, formation of de novo C16- and/or C24-Cer induces apoptosis in lymphoma cells in response to BcR triggering [27], in prostate carcinoma cells by androgen deprivation and MDA-7/IL-24 stimulation [39, 40], and in neutrophils by serum depletion [41]. Alternatively, apart from its biological effects as intracellular second messenger, Cer may also have biophysical effects through the stabilization of membrane lipid rafts affecting the localization of receptors and their function [42]. In relation to this possibility, it is of interest that membrane lipid rafts have been proposed to segregate proapoptotic from antiapoptotic IGFR1 signaling pathways [43]. Moreover, emerging evidence suggests that the redistribution of Apo2L/TRAIL death receptors to lipid rafts constitutes an important regulatory event for optimal activation of the apoptotic death program [44]. Obviously, the mechanisms for IGF1-mediated regulation of Apo2L/TRAIL-induced apoptosis are complex. Nevertheless, it is reasonable to propose that Cer-XIAP signaling constitutes a key pathway for Apo2L/TRAIL sensitization in ESFT cells in response to chronic IGF1 stimulation.

The proapoptotic capacity of IGF1 in the context of death ligand-induced apoptosis is not unique for ESFT cells. IGF1 has been reported to amplify Fas antibody-induced apoptosis in human osteoblasts, to stimulate TNF*α*-induced apoptosis in murine myoblasts and preadipocytes, and to enhance Apo2L/TRAIL-induced apoptosis in human colon carcinoma cells [43, 45–47]. 

We have shown that Apo2L/TRAIL-insensitive VH-64 cells are converted to sensitive after chronic IGF1 stimulation, whereas their sensitivity to DOX and VP16 is permanently blunted. The latter event occurred in spite of the inactivation of AKT. As far as the resistance to DOX and VP16 is concerned, the early activation of AKT by IGF1 could be the point of no return. However, alternative survival signaling pathways activated by IGF1 may as well promote resistance to chemotherapy, as discussed by Toretsky et al. [7]. By using cells transfected with AKT-MYR, these authors showed that constitutively activated AKT only partially accounted for the suppression of chemotoxicity in ESFT cells.

Moreover, we demonstrated that DOX- and VP16-induced cell death is not prevented by caspase inhibitor at a concentration (20 *μ*M) that readily prevented Apo2L/TRAIL-induced apoptosis. This result implies that the main mode of DOX- and VP16-mediated cell death at least in VH-64 cells is caspase independent. Our finding contrasts a study in which z-DEVD-fmk and z-VAD-fmk were shown to prevent the toxicity induced by VP16 in ESFT cells [48]. It should be noted that these authors employed caspase inhibitor at a concentration (100 *μ*M) that has been shown to inhibit noncaspase proteases in a nonspecific manner [49]. It is apparent from our findings that beyond its role in chemoresistance, IGF1 can circumvent this resistance and sensitize ESFT cells selectively to apoptosis triggered by Apo2L/TRAIL.

The physiological significance of our results has yet to be shown. Increasing evidence points to a pronounced regulatory role of IGF1 in the innate immune system [50]. As lymphohematopoietic factor, IGF1 has been reported to stimulate lymphocyte proliferation and survival, to accelerate cytokine-stimulated NK cell activity, and to support monocyte-derived dendritic cell maturation [51–53]. Recent studies have shown that lymphocyte recovery is an independent prognostic indicator for high-risk ESFT [54] and that ESFT cells are highly sensitive to killing by autologous dendritic cells and allogeneic NK cells in vitro and in vivo [55, 56]. Accordingly, the possibility that the combination of IGF1 and Apo2L/TRAIL in the long term may not only facilitate ESFT cell death directly but may also promote the induction of an anti-ESFT immune response in vivo is worthwhile considering.

Recently, interest has focused on the use of IGF1R antibody as potential therapy in ESFT patients [57]. Jürgens et al. [58] reported preliminary results from a phase I/II trial evaluating the efficacy of humanized IGF1R antibody as single agent, in which the treatment of 106 patients with advanced ESFT resulted in a modest overall response rate of 14.2% and median survival of 8.9 months. Considering our results demonstrating the apoptosis sensitizing effect of *α*-IR3 in long-term-treated ESFT cells in vitro, it is tempting to speculate that the clinical benefit of IGF1R antibody therapy could extend to more patients when it is combined with Apo2L/TRAIL-based regimens.

## 5. Conclusions

Despite improvements over the past two decades in the therapy of ESFT, the survival expectancy for patients with recurrent or metastatic disease is still low. It is recognized that IGF1 is associated with ESFT cell survival and chemoresistance. However, the present study paradoxically suggests that chronic IGF1 stimulation can bypass chemoresistance and support cell death in combination with Apo2L/TRAIL. Thus this study provides novel insight into the dynamic role of the IGF1-IGF1R system in regulating ESFT cell fate. Our finding showing that IGF1R antibody displays IGF1-mimitic actions may have important implications for the design of Apo2L/TRAIL-based therapies to treat refractory ESFT.
